# Crosstalk Between Ferroptosis and Cuproptosis in Intervertebral Disc Degeneration: Mechanisms, Therapeutic Targets, and Future Directions

**DOI:** 10.1002/jsp2.70080

**Published:** 2025-05-28

**Authors:** Zhongpan Li, Liangwei Wang, Xiaojun Wu, Rui Huang, Yi Yuan

**Affiliations:** ^1^ College of Integrative Chinese and Western Medicine Southwest Medical University Luzhou Sichuan China; ^2^ Department of Orthopedics Dazhou Integrated TCM & Western Medicine Hospital Dazhou Sichuan China

**Keywords:** cuproptosis, ferroptosis, future directions, intervertebral disc degeneration, mechanism

## Abstract

**Background:**

Intervertebral disc degeneration (IVDD) is a prevalent degenerative disease, with low back pain as its primary clinical symptom, imposing significant burdens on individuals and society. With the aging population, IVDD is becoming an inevitable challenge. Current research indicates that the pathogenesis of IVDD is primarily driven by aging, mechanical stress, cell death, and genetics, leading to the loss of nucleus pulposus and degradation of the extracellular matrix within the intervertebral disc.

**Objective:**

This review aims to explore the relationship between the mechanisms of ferroptosis and cuproptosis, two newly discovered modes of cell death, and their potential as therapeutic targets for IVDD.

**Methods:**

We conducted a comprehensive review of recent studies on ferroptosis and cuproptosis in IVDD, analyzing the mechanisms of these cell death patterns and their potential role in IVDD progression.

**Results:**

Ferroptosis and cuproptosis have been found to be closely related to IVDD. These cell death modes are implicated in the pathological processes of IVDD, suggesting a potential link between their mechanisms and the disease's progression.

**Conclusion:**

The mechanisms of ferroptosis and cuproptosis are closely related to IVDD, and these pathways may be potential targets for IVDD treatment, providing new directions for clinical treatment of IVDD and future research.

## Graphical abstract

GSH, oxidative stress, TCA cycle, autophagy, and copper are the intersection targets of ferroptosis and cuproptosis in IVDD.

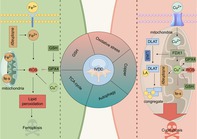

## 1 Introduction

Low back pain is one of the most common musculoskeletal problems in society and the leading cause of disability, experienced by nearly 80% of people [1, 2]. IVDD, as a condition with low back pain as the main symptom, presents a significant challenge to social health [3]. Current drug and surgical interventions for IVDD cannot solve its pathogenesis and pathological changes, resulting in limited efficacy and inability to reverse disease progression [4] Therefore, it is necessary to find new therapeutic directions to guide the treatment of IVDD.

At present, it is believed that the pathological changes of IVDD mainly include extracellular matrix (ECM) degradation, cell death, cell senescence, and inflammatory response [5]. Among them, during IVDD, intervertebral disc cells produce pro‐inflammatory factors, which further lead to cell death, autophagy, cell senescence, etc., and aggravate the degradation of the extracellular matrix [6]. In addition, these pro‐inflammatory factors recruit immune cells to further exacerbate IVDD [7, 8]. Understandably, research on intervertebral disc cells has attracted increasing attention. It has recently been found that both ferroptosis and cuproptosis are closely related to IVDD, and there is crosstalk between the two. Ferroptosis is a form of cell death characterized by an iron‐dependent excessive accumulation of lipid peroxides [9]. Cuproptosis is a form of mitochondrial cell death triggered by excess Cu^2+^ that differs from other cell death pathways, including apoptosis, ferroptosis, and necrotic apoptotic cells [10, 11].

Recent studies have identified the involvement of ferroptosis in IVDD, and inhibiting ferroptosis can effectively improve IVDD [12, 13, 14]. Similarly, studies have established that cuproptosis plays a critical role in the pathogenesis of IVDD by combining clinical cases with basic experiments [15]. Meanwhile, other studies have also found that metal regulatory transcription factor 1 (MTF1), a key gene for cuproptosis, is highly expressed in IVD and mainly exists in nucleus pulposus cells (NPCs) [16]. Serum copper ion levels in IVDD patients are higher than those in normal patients. It was also positively correlated with Pfirrmann classification [17]. It can be seen from the above studies that cuproptosis is closely related to IVDD and may be a potential therapeutic target.

It is worth noting that ferroptosis and cuproptosis are closely related in IVDD, with important interactions mainly at key nodes such as ion metabolism, mitochondria, and Glutathione (GSH) [18]. Therefore, we will focus on the interaction of ferroptosis and cuproptosis mechanisms and their relationship with IVDD in order to provide new strategies for clinical treatment of IVDD.

## 2 Intervertebral Discs

As a part of the whole spine, the intervertebral disc is composed of nucleus pulposus (NP), annulus fibrosus (AF), and cartilaginous endplate (CEP). One of the most important structures is the NP, which is located between AF and CEP and has some elasticity. As a weight‐bearing structure of the spine, the NP is able to evenly transfer stress to the annulus fibrosus, and this balancing effect effectively prevents the disc from being damaged by excessive local stress [19]. NPCs contain a large amount of collagen and proteoglycan [20], which help NPCs maintain a normal physiological state, mainly by regulating cell osmotic pressure, and this process involves multiple signaling pathways. The AF is composed of fibrocartilage and is plastic. AF is deformed by stress transmitted by the nucleus pulposus to cushion and absorb the load on the spine [21]. AF can effectively reduce the stress on the nucleus pulposus, vertebrae, and spine within a limited tolerable range. CEP is composed mainly of chondrocytes distributed in the hyalinoid cartilage matrix, and its main function is to transport nutrients such as sugar and oxygen to the disc through capillaries, which is extremely important for maintaining the structure and function of the disc [22].

## 3 Oxidative Stress and IVDD

The concept of oxidative stress was first proposed by Kirkman et al. in 1974. Oxidative stress is mainly a state of imbalance inside and outside the cell caused by excessive accumulation of oxidants (mainly reactive oxygen species), which exceeds the regulatory capacity of the antioxidant system [23]. Reactive oxygen species (ROS) mainly include superoxide anion radical (O_2_•^−^), hydroxyl radical (•OH), hydrogen peroxide (H_2_O_2_), and so on [24]. Similarly, some nitrogen‐containing compounds, such as nitric oxide and nitrogen dioxide, have similar oxidation effects to ROS and can also trigger oxidative stress [25]. It is understandable that when ROS production increases or ROS clearance decreases due to multiple factors, ROS will interact with macromolecules such as lipids, proteins, enzymes, nucleic acids, and further cause organelle dysfunction and cell death.

IVDD is a long‐term imbalance in extracellular matrix synthesis and decomposition, resulting in proteoglycan loss, disc height reduction, annulus fibrosus rupture, and cartilage endplate calcification. Studies have shown that inflammation [26], oxidative stress [23], mitochondrial dysfunction [27], abnormal mechanical load [28], and other factors regulate stromal metabolism, pro‐inflammatory phenotype, autophagy, aging, and cell death through IL‐1β, TNF‐α, and so on [29] to produce excess ROS, leading to ECM degradation and ultimately IVDD. In addition, ROS produced by oxidative stress during IVDD will activate the inflammatory signaling pathway leading to cell death, which will further promote the release of ROS, resulting in a positive feedback between inflammation and oxidative stress. In IVDD, Bcl‐2 family proteins lead to increased permeability of the mitochondrial outer membrane, resulting in apoptosis [30]. Activated NLRP3 inflammasome can cleave caspase‐1 precursor into caspase‐1‐induced pyrodeath [31, 32]; changes in the synthesis and decomposition of ECM [33], secretion of inflammatory factors [34], expression changes of autophagosomes and autophagy‐related markers [35], and cell senescence [36] are all closely related to oxidative stress. At the same time, relevant studies further clarified that oxidative stress can mediate ferroptosis [37, 38, 39] and cuproptosis [40] in the intervertebral disc, leading to IVDD.

## 4 Ion Metabolism in Ferroptosis and Cuproptosis

Iron is an essential trace element that plays an indispensable role in the body. Iron ions enter the cell mainly through transferrin (Tf), but can also rely on solute carrier family 39 member 14 (SLC39A14) and lactotransferrin (LTF) directly into the cell. After binding to iron ions, Tf enters the endosome via the transferrin receptor, where the STEAP reductase converts iron ions to ferrous ions [9, 41], and ferrous ions are transferred to the cytoplasm by divalent metal transporter 1 (DMT1) [42]. Some iron ions are stored in ferritin, some form unstable iron pools (LIP), and some are transferred out of the cell via membrane ferritransporters (FPN) [43]. When there is an excess of ferrous ions in cells, the excess ferrous ions will mediate the Fenton reaction to produce ROS [44]. On the one hand, ROS can further damage organelles such as mitochondria and cause further oxidative stress. On the other hand, ROS can cause polyunsaturated fatty acids on the cell membrane and plasma membrane (due to the presence of highly active hydrogen atoms, lipid peroxidation is more likely to occur) to be oxidized into lipid peroxides, and excessive lipid peroxides can cause cell rupture by destroying the stability of the lipid bilayer. This process requires the participation of acyl‐CoA synthetase long‐chain family members 4, lysophosphatidylcholine acyltransferase 3, lipoxygenases (LOX) and cytochrome P450 [9, 45, 46]. Intracellular GPX4 is able to reduce lipid peroxides to non‐toxic lipols [47], thereby avoiding cell death caused by lipid peroxide buildup. Cystine enters the cell via the SLC7A11 and SLC3A2 transporters, where it is converted into cysteine. Cysteine is one of the key substrates for GSH synthesis. Under the action of GSH, GPX4 reduces polyunsaturated fatty acid peroxide (PUFA‐OOH) to polyunsaturated fatty acid (PUFA‐OH) and inhibits ROS, thereby inhibiting ferroptosis.

**FIGURE 1:**
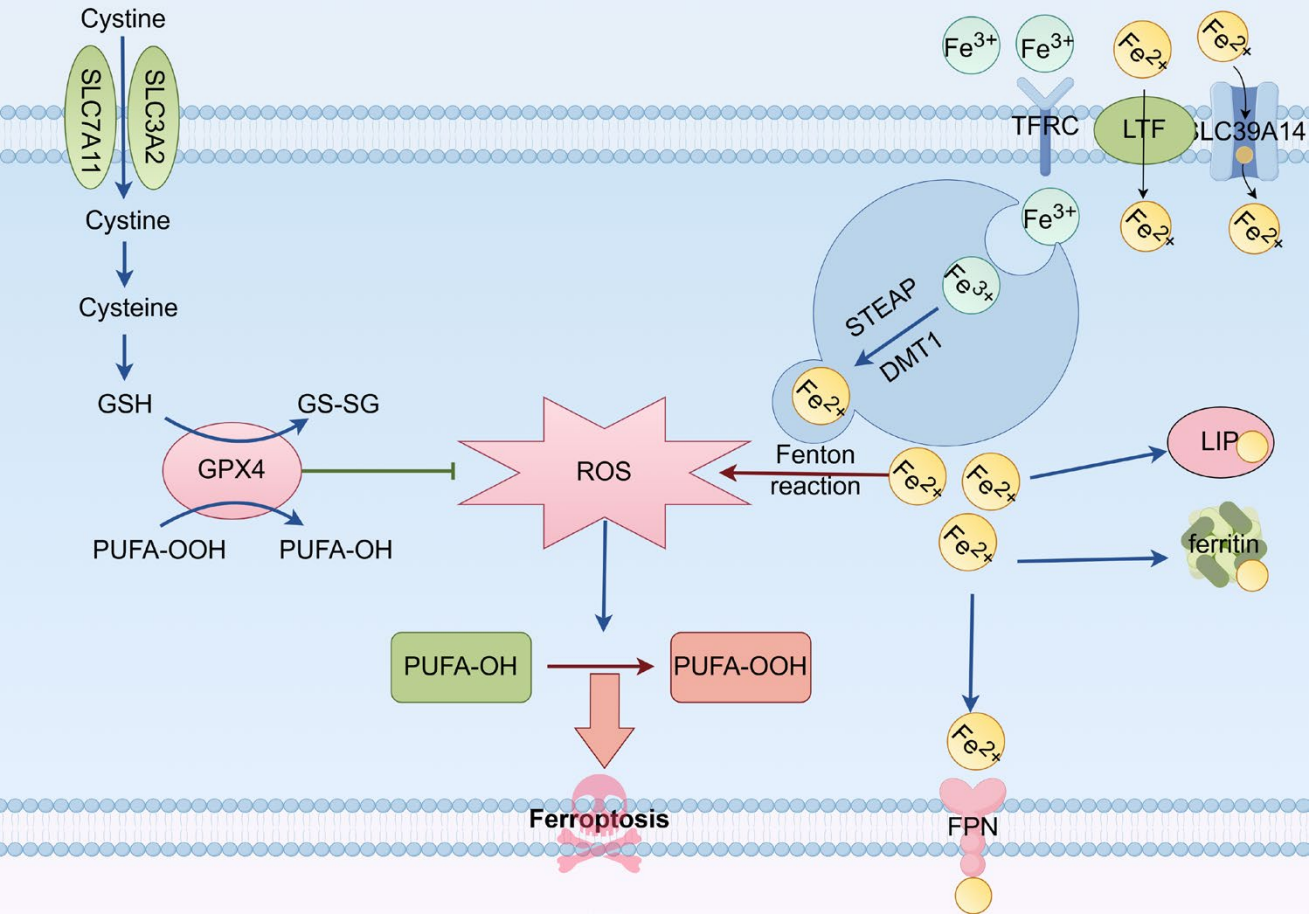
A brief mechanism for the occurrence of ferroptosis. Iron ions enter the cell through Tf, SLC39A14, and LTF. Tf enters the transferrin receptor to transport iron ions to the endosome, where the STEAP reductase converts iron ions to ferrous ions, which are transferred to the cytoplasm via DMT1. Some iron ions are stored in ferritin and LIP, and some are transferred out of the cell by FPN. Excessive ferrous ions in cells can mediate the Fenton reaction to produce ROS, which oxidizes the polyunsaturated fatty acids on the fine membrane into lipid peroxides, resulting in cell death. In the antioxidant system, cystine enters the cell via SLC7A11 and SLC3A2 transporters and is converted to cysteine to participate in the synthesis of GSH. Under the action of GSH, GPX4 inhibits ROS and thus inhibits ferroptosis.

In 2012, DIXON et al. defined non‐apoptotic cell death due to iron dependence as ferroptosis [48]. With the development of recent studies, lipid formation induced by ROS oxidation and the accumulation of lipid peroxides on the membrane have been found to be the main biochemical events of ferroptosis. In simple terms, iron ions in the cell can mediate the production of highly oxidizing ROS, resulting in a large amount of oxidation of the cell membrane and plasma membrane to produce lipid peroxides, and the intracellular GPX4 is insufficient to reduce lipid peroxides, leading to their accumulation on the membrane and causing cell death [49]. Figure 1 depicts the main mechanisms by which ferroptosis occurs.

**FIGURE 2:**
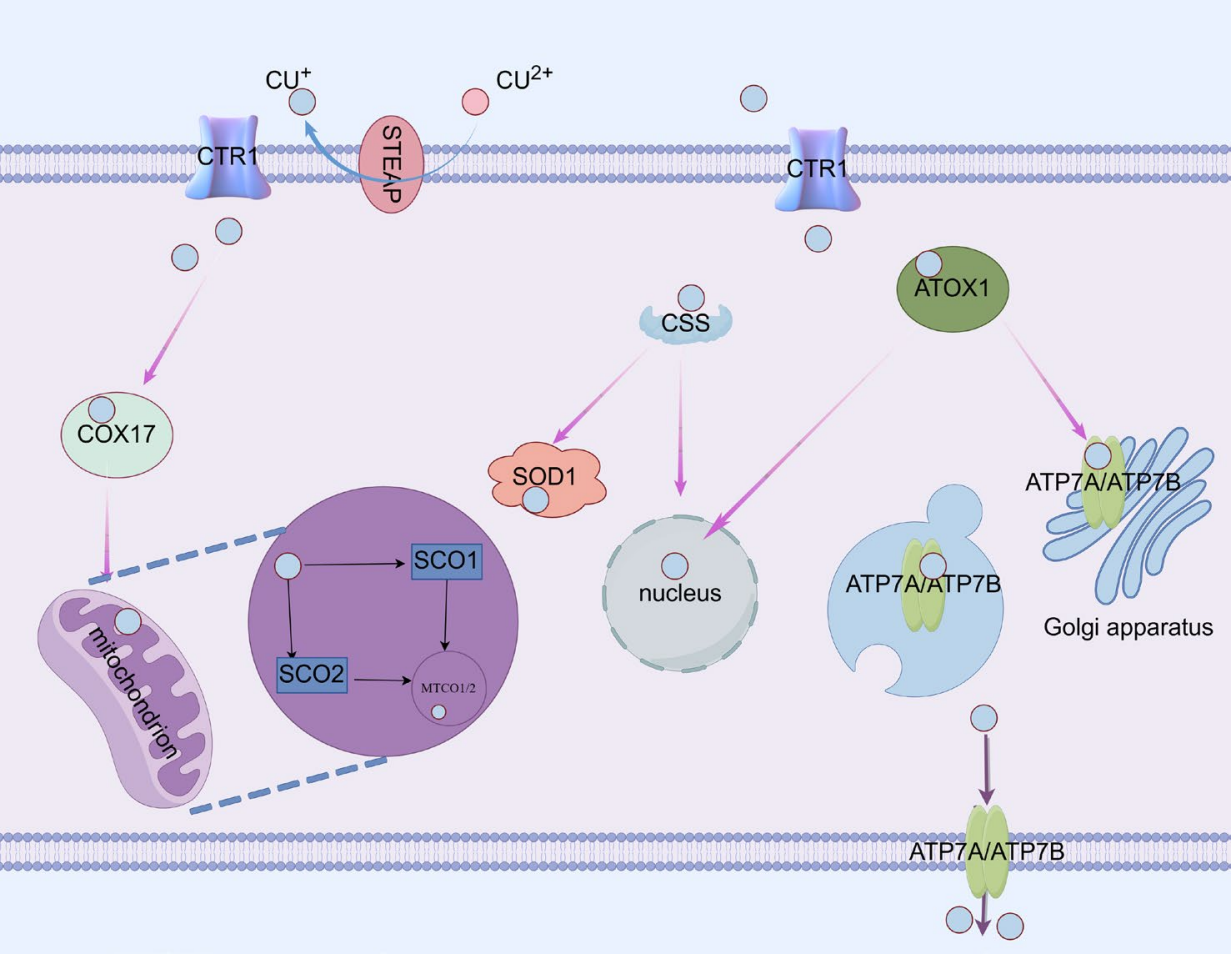
The diagram briefly describes the metabolism of copper ions. Copper ions are reduced to cuprous ions by STEAP, which are mediated into the cell by CTR1. Intracellular COX17 tranships cuprous ions to mitochondrial membrane proteins SCO1 and SCO2, which insert cuprous ions into MT‐CO2/COX2 and MT‐CO1/COX1, respectively. Cuprous ions are transported to the nucleus by ATOX1 and CCS. CCS can also pass Cu to SOD1. Cuprous ions can also be transported to ATP7A and ATP7B via copper's companion ATOX1. ATP7A and ATP7B can transport cuprous ions out of the cell.

Copper is an essential nutrient that enters the body through the duodenum and small intestine. Cupric ions in the small intestine need to be reduced to cuprous ions by metal reductase STEAP and duodenal cytochrome b (DCYTB) in order to be mediated by Cu transporters 1 (CTR1, SLC31A1) and CTR2 at the apex of intestinal epithelial cells [50]. Studies have shown that CTR1 expression decreases when Cu is excessive and increases when Cu is deficient [51, 52]. Intracellular cuprous ions enter mitochondria mainly through cytochrome oxidase copper chaperone cox17. First, COX17 transports cuprous ions to mitochondrial membrane proteins SCO1 (synthetic cytochrome C oxidase 1) and SCO2 (synthetic cytochrome C oxidase 2). Subsequently, SCO1 and SCO2 insert cuprous ions into MT‐CO2/COX2 (mitochondrial encoded cytochrome C oxidase II) and MT‐CO1/COX1 (mitochondrial encoded cytochrome c oxidase I), respectively [53]. Copper ions can also be transported to the nucleus by antioxidant 1 copper chaperone (ATOX1) and CCS(Cu chaperone for superoxide dismutase). CCS can also deliver Cu to superoxide dismutase 1 (SOD1) [54]. CCS increases when Cu content is low and decreases when Cu content is high [55]. In addition, monovalent copper ions can also be transported to ATP7A (ATPase copper transport α) and ATP7B (ATPase copper transport β) in the trans‐Golgi by copper companion ATOX1 [56]. Notably, as copper levels rise, ATP7A and ATP7B transport copper outward from the Golgi apparatus to facilitate the flow of excess copper extracellularly [57]. In the case of normal Cu content, ATP7A and ATP7B are located in the trans‐Golgi apparatus, and they transfer Cu from the cytoplasm into the trans‐Golgi apparatus. When there is too much Cu in the cell, ATP7A and ATP7B transfer from the trans‐Golgi apparatus to the vesicular compartment and fuse with the plasma membrane to expel Cu from the cell, and they re‐transfer to the trans‐Golgi apparatus when Cu content returns to normal levels [58]. We briefly describe the metabolism of copper ions in cuproptosis in Figure 2.

In 2022, Peter Tsvetkov et al. discovered a novel cell death pathway triggered by copper (Cu) named “cuproptosis” [11]. This type of cell death is not inhibited by inhibitors such as apoptosis, necrotic apoptosis, or ferroptosis, but copper chelating agents can rescue this type of cell death induced by copper [11]. Copper has been known to cause cell death since the 1980s [59]. Cuproptosis, as a newly discovered form of programmed cell death, is a mechanism by which excess intracellular copper binds to the fatty acylated components of the tricarboxylic acid (TCA) cycle to form the aggregation of Cu‐fatty acylated mitochondrial proteins, which reduces the generation of Fe‐S clusters and then triggers protein toxic stress, ultimately leading to cell death [60, 61]. It is characterized by a cell death caused by accumulation of lipoacylated proteins and loss of Fe‐S proteins by regulation of protein lipid acylation mediated by mitochondrial ferridoxin 1 (FDX1). Iron–sulfur (Fe‐S) is essential in mitochondrial respiration and affects the biological function of mitochondria [62, 63]. Lipoacylation is a highly conserved post‐translational modification in which all lipoacylated proteins participate in the TCA cycle. The TCA cycle is closely related to cell function and biological energy.

**FIGURE 3:**
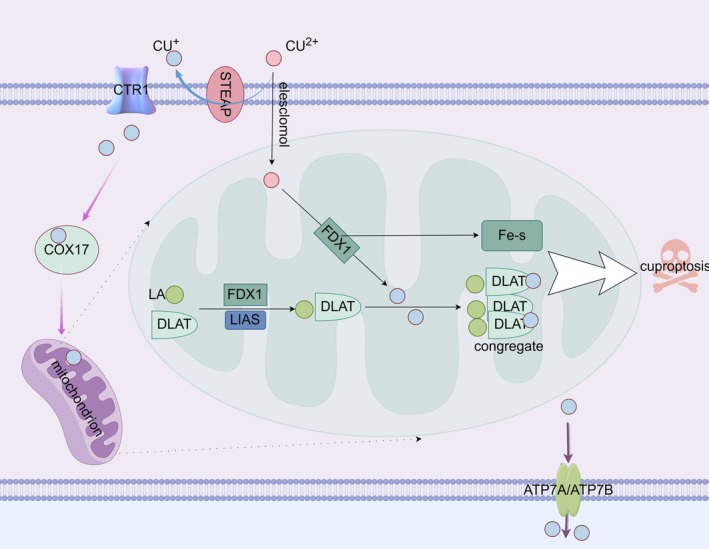
In cuproptosis, excess copper in the cell binds to the fatty‐acylated components of the TCA cycle, forming aggregates of Cu‐fatty‐acylated mitochondrial proteins. FDX1, a key regulator of cuproptosis, reduces copper ions to cuprous ions, promotes lipid acylation and aggregation of TCA‐cycle‐related proteins in mitochondria, and increases the binding of cuprous ions to lipoic‐acid‐containing proteins. Meanwhile, it promotes the loss of Fe‐S proteins. LIAS, involved in lipoacylation, is also a key regulator. Excess copper binds to FDX1‐mediated fatty‐acylated DLAT, causing abnormal DLAT aggregation, disrupting the TCA cycle, and triggering cytotoxic stress, ultimately leading to cell death.

In an initial study on cuproptosis, seven genes—FDX1, LIPT1, LIAS, DLD, DLAT, PDHA1, and PDHB—were found to be key regulators of cuproptosis [11]. FDX1 is a reductase known to reduce copper ions (Cu^2+^) to the more toxic cuprous ion (Cu^+^), and FDX1 is an upstream regulator of protein lipid acylation. The other six genes were protein lipid acylation pathway‐related molecules and targets. Specifically, copper ions are transported to intracellular partitions by binding with copper ionophores (e.g., elesclomol); in addition, SLC31A1 can also transport copper into the cell. Excess copper binds to FDX1‐mediated fatty acylated DLAT, leading to abnormal DLAT aggregation, further inducing TCA cycle disturbance and cytotoxic stress, eventually leading to cell death. In this process, FDX1, a key regulator of cuproptosis, reduces copper ions to cuprous ions while also promoting lipid acylation and aggregation of proteins associated with the TCA cycle in mitochondria and can further increase the binding of cuprous ions to these lipoic acid (LA) proteins. In addition, FDX1 promotes the loss of Fe‐S protein, and LIAS lipoic synthetase has been identified as a key regulator of cuproptosis due to its involvement in lipoacylation [64]. The mechanism by which cuproptosis occurs is depicted in Figure 3.

## 5 Crosstalk in Ferroptosis and Cuproptosis

### 5.1 Oxidative Stress

Oxidative stress is the imbalance between the oxidative and antioxidant systems inside the cell, which produces a large number of ROS. Notably, excess iron and copper ions both promote ROS production and further lead to DNA damage, mitochondrial dysfunction, lipid peroxidation, and protein modification, ultimately mediating different types of cell death [65, 66, 67]. Mechanically, both iron and copper can produce toxic hydroxyl radicals (OH) through the Fenton reaction, which can promote the oxidation system inside the cell. On the other hand, copper and iron promote GSH oxidation, a process that oxidizes GSH with antioxidant capacity to glutathione disulfide (GSSG) without antioxidant capacity [68].

### 5.2 Copper‐Induced Ferroptosis

The core of ferroptosis is iron metabolism, ROS accumulation, and lipid peroxidation caused by the Fenton reaction. It is worth noting that recent studies [69] have found that Elesclomol‐induced copper can also cause the Fenton reaction to produce a large number of ROS and further lead to ferroptosis. Elesclomol is a currently known copper‐chelating agent with clear efficacy in cancer treatment, but the mechanism is still unclear. In this study, the use of Elesclomol alone promoted the degradation of ATP7A and significantly inhibited the effect of copper, thus delaying the proliferation of CRC cells. Supplementation of copper with Elesclomol alone causes copper to accumulate in mitochondria due to ATP7A loss, which leads to the accumulation of reactive oxygen species and promotes the degradation of SLC7A11, which further exacerbates oxidative stress and leads to ferroptosis in CRC cells. During this process, excess copper induces the Fenton reaction, producing many ROS and triggering ferroptosis. At the same time, the degradation of SLC7A11 will lead to the reduction of intracellular GSH synthesis, and the activity of GPX4 will also be reduced, which will not be able to effectively reduce lipid peroxides and will also lead to the occurrence of ferroptosis. In addition, a 2023 study [70] found that copper was able to bind to the GPX4 protein's cysteine C107 and C148, increasing GPX4 ubiquitination and the formation of GPX4 aggregates. Subsequently, TAX1BP1 (TAX1‐binding protein 1) degrades GPX4 and produces ferritin autophagy mediated by copper.

### 5.3 Tricarboxylic Acid Cycle in Mitochondria

Mitochondria are relevant to biological processes due to their role in cellular energy supply, including ferroptosis and cuproptosis. Ferroptosis is characterized by reduced mitochondrial volume, increased membrane density, outer membrane rupture, and ridge disappearance, along with decreased ATP synthesis and DNA damage caused by mitochondrial damage [71]. Mitochondria are the main suppliers of intracellular ROS and are also the key sites of ferroptosis. Similarly, when cuproptosis occurs, mitochondrial contraction and membrane rupture occur [72]. Remarkably, the tricarboxylic acid cycle acts as a bridge between ferroptosis and cuproptosis in the mitochondria.

Glutamine breakdown as an anaerobic pathway of the mitochondrial TCA cycle can increase lipid peroxidation to promote ferroptosis, and blocking the TCA cycle or glutamine loss improves cystine depletion or Erastin‐induced ferroptosis [73]. The role of the mitochondrial TCA cycle in cuproptosis is more self‐evident, and protein lipoylation induced by four proteins of the TCA cycle (DBT, GCSH, DLST and DLAT) is a key link in cuproptosis [10]. Copper causes cuproptosis by aggregating lipoacylated proteins and causing loss of Fe‐S cluster proteins, which destroys mitochondrial integrity [10, 60].

Meanwhile, the mitochondrial electron transport chain (ETC) plays a central role in initiating ferroptosis by influencing mitochondrial membrane potential, lipid peroxides, and ferroptosis [73]. In simple terms, the electron transport chain of mitochondria mainly provides energy. Notably, cuproptosis was inhibited using electron transport chain complex inhibitors I and III, as well as mitochondrial pyruvate transporter inhibitors [10]. In addition, when mitochondrial energy is severely depleted, AMP‐activated protein kinase (AMPK) is activated to promote cuproptosis [11] It is not difficult to see from the above that mitochondrial TCA is the convergence point of ferroptosis and cuproptosis.

### 5.4 GSH

In 1888, De‐Rey Pailhade discovered GSH in food, which was officially named GSH in the 20th century [74]. GSH is a widespread intracellular tripeptide synthesized from glutamic acid, cysteine, and glycine. Its main antioxidant effect plays an important role in cell homeostasis, and it has been reported that GSH can directly clear several ROS and RNS [75, 76]. The functional group of GSH is the mercaptan of the cysteine part, and cysteine is the rate‐limiting substrate of GSH biosynthesis. The precursor of cysteine is reduced to cysteine after entering the cell through cystine/glutamate antiporters on the cell membrane. In addition, GSH itself can release its own cysteine through gamma‐glutamyltranspeptidase (gamma‐GT) and dipeptidase (DP). Cysteine forms GSH with two other amino acids under the action of glutamate‐cysteine ligase (GCL) and glutathione synthetase (GSS). GSH mainly exists in the cell as a reduced form, and a small amount of GSH is oxidized to GSSG by ROS and regenerated into GSH by GSSG reductase and NADPH.

In studies of ferroptosis, GSH is primarily thought to act as an antioxidant, providing cysteine residues and electrons to GPX4, preventing lipid peroxidation and ferroptosis [77]. However, GSH also binds to copper as an intracellular copper chelator to reduce the aggregation of lipoacylated proteins, thereby inhibiting cuproptosis [78]. In addition, copper can promote ferroptosis by stimulating autophagy degradation of GPX4 [70].

Interestingly, GSH showed inhibitory effects on both ferroptosis and cuproptosis, suggesting that GSH may play a key role in mediating the co‐regulatory relationship between these processes. A recent study [79] showed that ferroptosis inducers sorafenib and erastin enhanced copper ionophore‐induced cell death in primary liver cancer cells. In addition, the combination of sorafenib or erastin with elesclomol treatment significantly increased the accumulation of lipoacylated DLAT in liver cancer cells, suggesting that these inducers not only induced ferroptosis but also promoted cuproptosis. Similarly, BSO is a GSH synthesis inhibitor known to induce ferroptosis [18], and recently found to induce cuproptosis as well [11]. An RNA sequencing study found that miR‐15a‐5p was a potential biomarker in IVDD, and it happened that copper cancer‐related genes, ferroptosis‐related genes, and oxidative stress‐related genes were all potential targets of miR‐15a‐5p.

### 5.5 Autophagy

**FIGURE 4:**
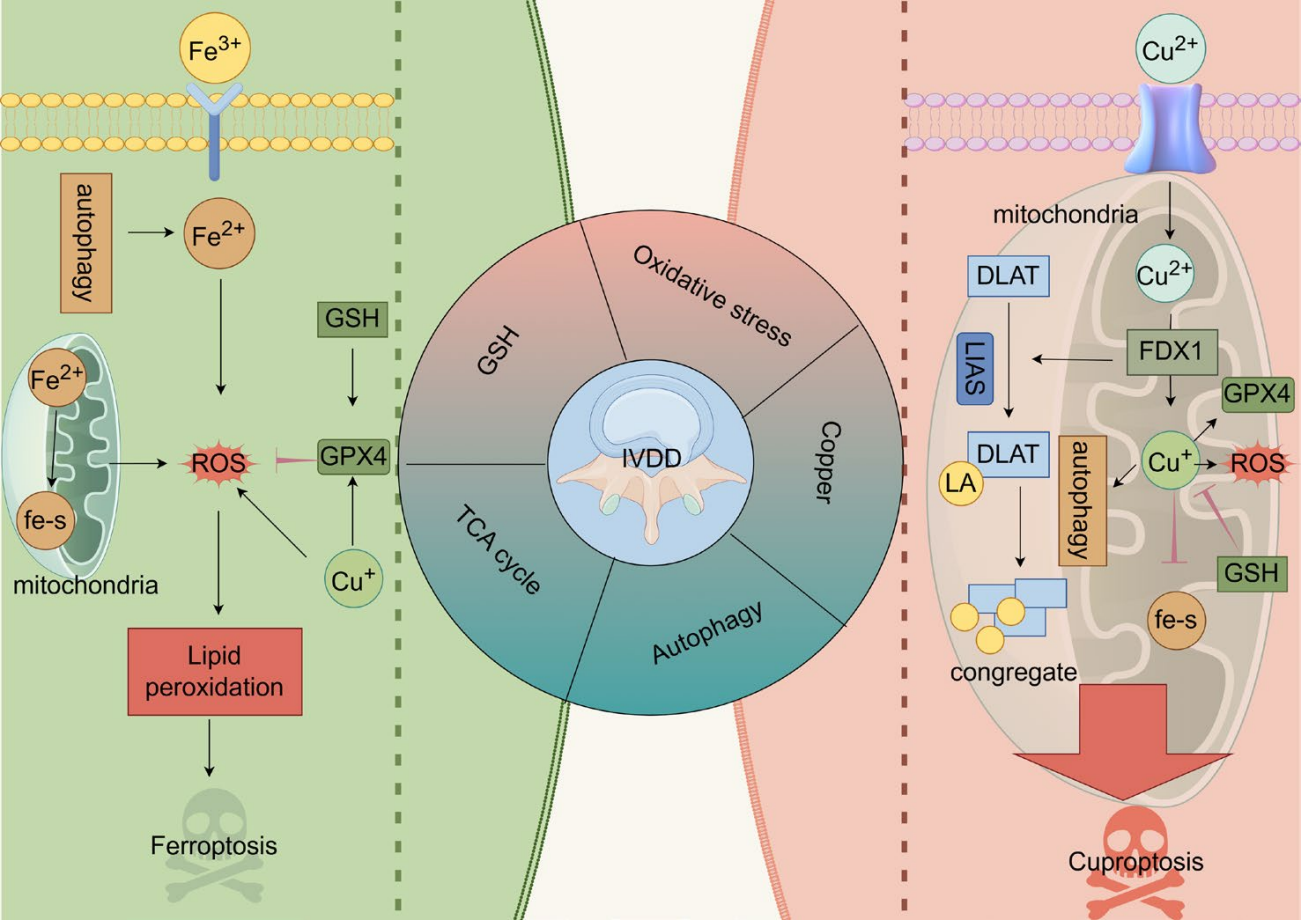
The figure combines ferroptosis and cuproptosis in IVDD and identifies five key targets of GSH, oxidative stress, TCA cycle, autophagy, and copper.

Autophagy can regulate ferroptosis by interfering with signals related to ferroptosis. Numerous studies have shown that the release of iron ions in ferritin by regulating autophagy, including ferritin autophagy, clock autophagy, and adipose autophagy, can induce and promote ferroptosis [80, 81, 82, 83]. Similarly, copper not only induces cuproptosis but also drives ferroptosis by affecting the degradation of GPX4 [71]. Similarly, excess intracellular copper sequestosome 1 (a lipoautophagy associated protein) regulates the AMPK‐MTOR pathway to induce autophagy [84]. Biogenic analysis found that FDX1 was positively correlated with autophagy marker gene expression, suggesting a potential crosstalk between FDX1‐mediated cuproptosis and autophagy [85]. However, a link between autophagy and ferroptosis and cuproptosis, respectively, has been established, but the correlation between ferroptosis and cuproptosis through autophagy needs further research. We depict in Figure 4 the five key interacting targets of ferroptosis and cuproptosis in IVDD described above.

## 6 Ferroptosis and Cuproptosis in IVDD

In the process of IVDD, ferroptosis and cuproptosis play a very important role, and there are complex correlations between them, which jointly affect the occurrence and development of IVDD. A large number of studies have fully confirmed that ferroptosis is involved in the pathological process of IVDD. As an important factor in the occurrence and development of IVDD, oxidative stress will promote the accumulation of ROS in large quantities, and ROS will further release iron ions from ferritin through autophagy to promote ferroptosis [86]. Excess ferrous ions produce more ROS through the Fenton reaction, and these ROS attack polyunsaturated fatty acids on the cell membrane, resulting in intensified lipid peroxidation. During IVDD, due to increased oxidative stress, the activity of GPX4 is inhibited, and lipid peroxides cannot be cleared in time, resulting in ferroptosis of cells. The occurrence of ferroptosis can lead to impaired intervertebral disc cell function, reduced extracellular matrix (ECM) synthesis, and promote ECM degradation, further aggravating IVDD.

According to a team of researchers from the Department of Orthopedics, Qilu Hospital, Shandong University [87], Piezo1, a mechanically sensitive ion channel, is up‐regulated during IVDD, directly promoting iron inflow, altering biomarkers related to iron metabolism, affecting GPX4 expression, and causing changes in intracellular Fe^2+^ as well as iron metabolism and iron‐death protein levels. Finally, ferroptosis is induced and the IVDD process is promoted.

Cuproptosis is also strongly associated with IVDD. Experimental studies in combination with machine learning and clinical cases have confirmed that cuproptosis plays a crucial role in the pathogenesis of IVDD [15]. It was found that serum copper ion levels in patients with IVDD were higher than those in the normal population and were positively correlated with Pfirrmann classification, suggesting that copper ions played an important role in the progression of IVDD [17]. In the study of oxidative stress‐induced cuproptosis [40], by establishing in vitro and in vivo models to study cuproptosis in IVDD and the mechanism of interaction between oxidative stress and copper sensitivity in NPCs, it was confirmed that oxidative stress up‐regulates FDX1 expression and copper flux by promoting SP1‐mediated CTR1 transcription. TCA cycle‐related protein aggregation and copper flux increase.

Ferroptosis and cuproptosis do not exist in isolation in IVDD, and there is significant crosstalk between them. First of all, copper ions can induce ferroptosis. In addition, copper ions can directly bind to GPX4 protein, promote GPX4 ubiquitination and aggregation formation, and then degrade GPX4 through the autophagy pathway, resulting in ferroptosis. More succinctly, oxidative stress, mitochondrial TCA cycling, GSH, and autophagy connect the two as intersections between ferroptosis and cuproptosis.

## 7 Summary and Outlook

In summary, IVDD is a complex degenerative disease; its pathogenesis involves many factors, and ferroptosis and cuproptosis, as newly discovered cell death modes, play an important role in the occurrence and development of IVDD. Ferroptosis and cuproptosis each have unique metabolic pathways and regulatory mechanisms, and there is extensive and complex crosstalk between them, which affects each other through multiple links such as oxidative stress, the mitochondrial TCA cycle, GSH, and autophagy. In IVDD, the abnormal activation of ferroptosis and cuproptosis can lead to a series of pathological changes such as impaired intervertebral disc cell function, extracellular matrix degradation, and increased inflammatory response, which jointly promote the progression of the disease.

At present, although some progress has been made in the study of ferroptosis and cuproptosis in IVDD, there are still many unknown areas to explore. Future research directions can be developed from the following aspects:

At the basic research level, further exploring the molecular regulatory mechanism of ferroptosis and cuproptosis in IVDD, studying how to accurately regulate the occurrence process of ferroptosis and cuproptosis, and searching for key targets that can specifically interfere with these two cell death modes are of great significance for the development of new therapeutic strategies for IVDD.

In terms of clinical application, based on the in‐depth understanding of the mechanism of ferroptosis and cuproptosis, the development of therapeutic drugs targeting ferroptosis or cuproptosis is essential. The magnetic hydrogel developed by the Soochow University team(89) can adsorb excess iron ions, reshape iron metabolism, and inhibit ferroptosis, which provides ideas for the development of related drugs and can be optimized in the future on this basis.

In addition, considering the complex relationship between ferroptosis and cuproptosis and other cell death modes (such as apoptosis, necrotic apoptosis, etc.) as well as cell biological processes (such as cell senescence, autophagy, etc.), future studies should comprehensively consider multiple factors to build a more comprehensive pathogenesis model of IVDD. This will contribute to the development of more accurate and effective comprehensive treatment programs and bring new hope to patients with IVDD.

Ferroptosis and cuproptosis have opened up a new direction for the study of IVDD. With the in‐depth understanding of its mechanism and the continuous development of related treatment strategies, it is expected to bring breakthroughs in the clinical treatment of IVDD, improve the quality of life of patients, and reduce the social medical burden.

## Author Contributions

Zhongpan Li designed the entire manuscript and prepared Figures 1, 2, 3, 4. Liangwei Wang participated in the writing process, Xiaojun Wu reviewed the literature and shared the writing drafts. Rui Huang reviewed the literature and shared the writing drafts. Yi Yuan performed the final review of the manuscript. All authors have read and approved the final manuscript.

## Ethics Statement

The authors have nothing to report.

## Conflicts of Interest

The authors declare no conflicts of interest.

## Data Availability

The authors have nothing to report.
